# Metabolic relationship between diabetes and Alzheimer's Disease affected by Cyclo(His-Pro) plus zinc treatment

**DOI:** 10.1016/j.bbacli.2016.09.003

**Published:** 2016-10-02

**Authors:** Moon K. Song, David S. Bischoff, Albert M. Song, Koichi Uyemura, Dean T. Yamaguchi

**Affiliations:** a VA Greater Los Angeles Healthcare System, 16111, Plummer Street, North Hills, CA 91343; b UCLA School of Medicine, 1O833 Le Conte Avenue, Los Angeles, CA 90095; c Kaiser Permanente Medical Center, 13651 Willard Street, Panorama City, CA 91402

**Keywords:** Diabetes, Alzheimer's Disease, Metabolic disease, Cyclo(His-Pro), Zinc, Insulin degrading enzyme

## Abstract

**Background:**

Association of Alzheimer's Disease (AD) with Type 2 Diabetes (T2D) has been well established. Cyclo(His-Pro) plus zinc (Cyclo-Z) treatment ameliorated diabetes in rats and similar improvements have been seen in human patients. Treatment of amyloid precursor protein (APP) transgenic mice with Cyclo-Z exhibited memory improvements and significantly reduced Aβ-40 and Aβ-42 protein levels in the brain tissues of the mice.

**Scope of review:**

Metabolic relationship between AD and T2D will be described with particular attention to insulin sensitivity and Aβ degradation in brain and plasma tissues. Mechanistic effect of insulin degrading enzyme (IDE) in decreasing blood glucose and brain Aβ levels will be elucidated. Cyclo-Z effects on these biochemical parameters will be discussed.

**Major conclusion:**

Stimulation of IDE synthesis is effective for the clinical treatment of metabolic diseases including AD and T2D.

**General significance:**

Cyclo-Z might be the effective treatment of AD and T2D by stimulating IDE synthesis.

## Highlights

• Alzheimer's Disease is called Type 3 Diabetes as associated with Type 2 Diabetes.
• Insulin degrading enzyme treats both Alzheimer's Disease and Type 2 Diabetes.
• Cyclo(His-Pro) plus zinc treats both Alzheimer's Disease and Type 2 Diabetes.

## 1 Introduction

Alzheimer's Disease (AD) is the most common form of dementia culminating in the gradual accumulation of amyloid-beta (Aβ) protein into microscopic “plaques” and the twisting of tau proteins into strands of dead and dying neurons. It is also characterized in the early stages with defects in inflammation and oxidative stress [1]. Inflammation is especially important as it occurs in pathologically vulnerable regions of AD and can influence AD development. Individuals suffering from AD exhibit several behavioral symptoms including: confusion, disorganized thinking, memory loss, impaired judgment, and disorientation. In the final stages, they lose the ability to communicate, fail to recognize loved ones, and become bed-bound, which is ultimately fatal. About 5 million Americans were afflicted with AD in 2013 [2] and this number is projected to be 14 million in the USA alone by the year 2050. Worldwide, nearly 44 million individuals are currently afflicted with this disease [3]. The cost of caring for AD patients in the US was estimated to be $226 billion in 2015 with the global cost for caring estimated to be $605 billion [4].

Diabetes, both Type 1 and 2, are also major health concerns. The pathology of Type 1 diabetes (T1D) is insulin deficiency with no β-cell response to glucose. Type 2 Diabetes (T2D) starts with hyperinsulinemia, but this condition deteriorates during the progression of diabetes until the patients can no longer produce insulin. Many T2D patients have sufficient β-cell reserve to maintain a relatively euglycemic or normal blood glucose state; however, in the advanced disease state, insulin production by the pancreatic β-cell is reduced, and insulin administration is required. More than 90 percent of all diabetic patients have T2D, also known as adult onset diabetes. Over 29 million Americans (9.3% of the total population) were reported to be diabetic in 2012 according to the National Diabetes Statistics Report (2014) published by the National Center for Chronic Disease Prevention and Health Promotion, which was an increase from 25.8 million in 2010. Extrapolating from this increase, it is projected that more than 30 million Americans will be diagnosed as diabetic in 2016. Furthermore, 25.9% of Americans over age 65 are diabetic and 86 million were considered pre-diabetic in 2012. Worldwide diabetics increased from 108 million in 1980 to 422 million in 2014 [5] representing 6.6 ± 3.8% of the world population in the years 2000–2012 [6]. International Diabetes Federation Report estimates are lower but still significant showing that 2.8% of the world population are currently diabetic and estimating 4.4% or 366 million diabetics by 2030 [7]. Diabetes ranks as the seventh leading cause of death in the US (67,071 deaths in 2010) with a cost of care exceeding $245 billion in the US alone (2012). Thus, AD and diabetes represent specific health problems and significant medical expenses for care in the aged population.

## 2 Clinical manifestations

### 2.1 Alzheimer's Disease

Alzheimer's Disease (AD) is a neurodegenerative disease in which the brain actually shrinks as more brain cells die. Signs of the disease are initially behavioral with memory defects and the inability to recognize faces or objects, problems in communication, impaired judgment and reasoning, and changes in personality. Several cellular and molecular mechanisms (oxidative stress, mitochondrial dysfunction, inflammation, proteotoxicity, and altered gene expression) are involved in AD [8]. Although some genetic factors may contribute, sporadic form of AD mainly occurs after the age of 65. According to the Alzheimer's Association report in 2015, the annual new cases of AD in Americans of 65–74 years old were 2/1000 to13 new cases/1000, respectively, and 39 new cases/1000 in age 85 and older [9]. Many of the oldest AD patients are also afflicted with cerebrovascular disease and typically experience brain inflammation. These facts suggest that AD is an adult onset metabolic disease experienced as aging progresses. Some researchers believe that aging which influences the oxidative and inflammatory states of the brain is the most important risk factor for AD [10]. Oxidative stress with toxic oxygen free radicals are also regarded as pathological abnormalities. Currently, the apolipoprotein E (APOE) gene is the only gene that is related to late onset AD; although, correlation with the development of the disease in patients and genetic mutation is weak. The apolipoprotein E4 (APOEε4) allele is present in about 25–30% of the general population and 40% of AD, which also runs in the family. Individuals carrying the ε4 allele are at increased risk of AD compared to those carrying the more common ε3 allele [11]. APOEε4 is a major cholesterol carrier that supports lipid transport and injury repair in the brain. Mild cognition impaired (MCI) subjects have higher APOEε4 levels compared to non-MCI subjects [12].

The most significant risk factor for AD is unhealthy lifestyle choices. Cigarette smoking and obesity are associated with an increased risk for the development of both vascular and non-vascular dementia in the aging population [13]. According to the Alzheimer's Association report of 2016, the risk of getting AD increases if a family member also has the disease. Aside from the genetic consideration of the APOEε4 allele which may be familial, this risk may also be due to lifestyle similarities in family members in regards to energy consumption or expenditure, such as overeating of high fat foods, or lack of exercise, and stress due to family or career obligations. Other identified risk factors are high blood pressure, high cholesterol, and head trauma. Minimizing these lifestyle risks factors may be helpful in delaying and possibly treating AD by reversing metabolic abnormalities though healthy living.

### 2.2 Type 2 Diabetes

Type 2 Diabetes (T2D) is the adult onset form of diabetes. T2D begins with insulin resistance in the absence of any clinical symptoms; however, over time insulin sensitivity gradually diminishes, and eventually reaches a point where insulin signaling does not work even though the body is producing increased levels of insulin. When insulin resistance reaches this severe stage, pancreatic beta cells no longer produce enough insulin to overcome the lack of insulin signaling, and blood glucose levels rise ultimately resulting in diabetes. Initially T2D symptoms are subtle with the patient exhibiting thirst and frequent urination due to the high levels of osmotically active glucose in the blood which serves to pull fluid from the tissues. Subsequent volume depletion can result in dizziness and orthostatic hypotension and syncope as well as non-specific symptoms such as lethargy. Fluid may also be removed from the eye lenses, affecting its ability to focus, and resulting in blurred vision. Diabetics may also exhibit tiredness due to cells being deprived of glucose. Even though diabetics tend to eat more to relieve hunger, they tend to lose weight, since they metabolize alternate fuels stored in muscle and fat due to the inability to metabolize glucose. The most important diabetes related clinical complications are neuropathy, nephropathy and retinopathy. Diabetic neuropathy is a wide range of dysfunctions. The most common form of it is *distal symmetric polyneuropathy* affecting somatic sensory, motor nerve and autonomic nervous system. Although the exact mechanisms are not clear, T2D patients with lower extremity ulceration exhibited increased risk of severe retinopathy [14]. Diabetic nephropathy is observed in the presence of persistent proteinuria in sterile urine of patients with elevated blood pressure. Twenty five to 50% of diabetic patients develop disease and require kidney dialysis or transplantation. Among US adults with diabetes from 1988 to 2014, prevalence of diabetic kidney disease did not change, while prevalence of albuminuria and eGFR declined [15]. Diabetic retinopathy is the most common cause of blindness. Classically, diabetic retinopathy is divided into three stages; back ground, preproliferative, and proliferative retinopathy. Background retinopathy may be reversible if normal or near normal blood glucose levels are maintained [16]. Preproliferative retinopathy is increasing retinal ischemia due to capillary nonperfusion. Proliferative diabetic retinopathy is the presence of newly formed blood vessels or fibrous tissue arising from the retina and extending along the inner surface. Combined tractional and rhegmatogenous retinal detachment is rare (7–35%) but a serious complication in this disease [17].

### 2.3 Common clinical manifestations between Alzheimer's Disease and Type 2 Diabetes

Alzheimer's Disease (AD) and Type 2 Diabetes (T2D) are very serious health problems with vastly different symptoms yet share a complex and linked mechanism. T2D is a disease in which the body fails to use insulin properly and progresses to a state where the beta cells of the pancreas produce very little insulin. On the other hand, AD is a neurodegenerative disease of the brain with Aβ deposit and tau protein tangles in brain tissues. Although not all research results are in agreement, the majority of the data suggests that diabetics, specifically T2D subjects, are at much higher risk of developing AD than normal subjects. Elderly people with diabetes have an especially increased risk of mild cognitive impairment (MCI) [18] and an association of diabetes with MCI varies with amyloid subtype (amnestic) MCI and nonamnestic MCI) and number of domains, and with the sex of the patient, with females being more susceptible than males [19]. Diabetes as a significant risk factor for dementia or AD has been clearly established by studies in the US [20] and several other countries (Taiwan [21], China [22], Japan [23], and Finland [24]). T2D has also been associated with an increased risk of vascular dementia [25], and pre-diabetic insulin resistance is a risk factor for AD pathology and reduced memory function [26]. Some studies indicate that improving T2D can delay or prevent AD pathology [27]; and therefore, prevention and control of diabetes may reduce the risk of MCI and AD later in life [28]. A significant association between diabetes and AD has also been reported by linking insulin and glucose metabolism load to β-amyloid levels in AD [29]. In this study, it was demonstrated that oral glucose intake significantly increased plasma Aβ levels in AD patients compared to non-AD controls. These data suggest that T2D and AD originate from a common cause (lifestyle mismanagement) and share common pathological conditions such as insulin sensitivity, metabolism, or deficiency. Interestingly, a common enzymatic link between the two diseases is found in the insulin degrading enzyme (IDE). IDE is a zinc protease and the most well known enzyme that degrades both insulin and extracellular and intracellular Aβ [30]. Zinc deficiency which has been noted in both AD and T2D patients [31], [32], [33], which causes IDE deficiency since zinc is an absolute requirement for IDE synthesis. Since IDE deficiency contributes to the induction of some forms of AD [34], [35], increasing degradation of extracellular Aβ by IDE or similar enzymes would be beneficial for the possible prevention and/or treatment of AD [36]. Degradation of internalized insulin also improves insulin sensitivity, implicating a potential treatment of diabetes [37].

## 3 Pathophysiology

### 3.1 Alzheimer's Disease

Alzheimer's Disease (AD) is a progressive form of dementia with loss of neurons and the presence of two neurological hallmarks: extracellular amyloid plaques and intracellular neurofibrillary tangled tau proteins in the brain. AD research has been focused on the use of animals to both improve our understanding of the pathophysiology of AD and to test novel therapeutic approaches. Unfortunately, the animal research thus far has not been successfully translated into therapeutic treatment(s) for human patients. Amyloid plaque formation plays a pivotal role in AD pathogenesis [38]. Accumulation of β-amyloid peptides (Aβ) in the brain is the first critical step in the pathogenesis of AD [39] and is mainly due to the aggregation of Aβ which is cleaved sequentially from amyloid precursor protein (APP) by two enzymes, β-secretase and γ-secretase. The order of cleavage is very important since if α-secretase cleaves APP first, then Aβ plaques are not formed. Neurofibrillary tangles result from aggregation of the tau protein, a component of the internal structure of the nerve cells that is associated with the microtubules and functions to facilitate neuronal transport. If tau protein is prevented from binding to the microtubules, it self-aggregates into tangles, promoting microtubule disassembly, and inhibiting neuronal transport. Tauopathy is initiated by forming neurofibrillary tangles, neuritic plaques, and neuropil threads [40]. Excessive phosphorylation of tau has also been shown to contribute to tangle formation [41] and prevents neuronal cell function. In AD, brain synapses are disrupted by the Aβ plaques and tau tangles, damaged neurons die. Since neurons in the brain connect and communicate at synapses where neurotransmitters carry information from one cell to the other; the result is disruption of the brain's communication network.

### 3.2 Type 2 Diabetes

In T2D, the patient is unable to process insulin signaling correctly, making the body insulin-resistant, which is the inability of cells to respond adequately to normal levels of insulin within the liver, muscle, and fat tissues. In the liver, insulin suppresses glucose release, but in the setting of insulin resistance, the liver inappropriately releases glucose into the blood. However, not all people with insulin resistance develop diabetes, since an increase of insulin secretion by pancreatic beta cells is frequently seen as the disease progresses. Initially, the pancreas makes more and more insulin to keep up with the insulin demand due to the loss of the ability of muscle and fat tissues to utilize insulin effectively. Diabetes occurs when the pancreas loses its ability to produce enough insulin to compensate for the insulin resistance and as a result, blood sugar levels are no longer kept in control. The proportion of insulin resistance versus beta cell dysfunction (insulin secretion) differs among individuals, with some having primarily insulin resistance and only a minor defect in insulin secretion; whereas, others have slight insulin resistance and primarily a lack of insulin secretion. Many people think of T2D as the more common form of diabetes affecting adults, but it has become an increasingly serious childhood problem as obesity rates are alarmingly increasing due to consumption of high energy food containing high fat and sugar demonstrating that the type of food consumed, activity level, genetics, and other external factors have significant roles as well. While the specific cause of T2D is still not clearly known, researchers have identified several potentially important mechanisms associated with T2D and insulin resistance: increased breakdown of lipids in fat cells, resistance to and lack of incretin, high glucagon levels in the blood, increased retention of salt and water by the kidneys, and inappropriate regulation of metabolism by the central nervous system.

#### 3.2.1 Lifestyle effects of Type 2 Diabetes

The main lifestyle cause of T2D is excessive consumption of food (hyperphagia), due to the abundance of processed food in modern life. The number of people with T2D and those considered obese (obesity is a symptom of insulin resistance) has increased over the last decade. In countries where meat consumption is low, such as in Asian countries where there is less consumption of protein but a high carbohydrate diet, diabetes develops without showing obesity. It is preferable to consume an animal protein diet since plant protein sources such as phytic acid from soy bean are difficult to digest for producing needed amino acids in the body. Thus, in Asian countries, the main protein sources are soy proteins and other vegetables and T2D can develop without high obesity. In Westernized countries, where meat consumption is high, obesity, lack of exercise, and poor diet all contribute to the development of T2D in obese subjects afflicted with insulin resistance and mild or no diabetes. The high protein intake (meat) initially allows the fat cells to grow rapidly without developing diabetes; however, the subjects become obese and then later develop diabetes. Experts suggest a link between people who carry their weight more prominently in the abdomen and a higher likelihood of developing T2D [42]. High sugar intake isn't necessarily a precursor for T2D, but eating and drinking excessive amounts of carbohydrates make it easier to become overweight when consuming high proteins. Failing to be active and not getting the recommended amount of exercise also contributes to insulin resistance and an overall less healthy system, which eventually triggers T2D. People who are obese tend to be less active and more likely to not eat a healthy balanced diet rich in vitamins and nutrients that the body needs to function at top capacity.

#### 3.2.2 Genetics of Type 2 Diabetes

While an unhealthy lifestyle is a major contributor, not every overweight person will develop T2D and some slender, fit people will be born with an inherent likelihood to eventually develop T2D. Not only can hereditary factors impact the likelihood of an individual becoming overweight, but researchers have found that certain DNA alleles directly affect insulin production and secretion. The National Institute of Health states that having two copies of the transcription factor 7-like 2 (TCF7L2) gene makes an individual 80% more likely to develop T2D over his/her lifetime [43]. There are no other specific genetic abnormalities linked to the inheritance of diabetes. The increasing number of cases of childhood obesity is not simply because these children are not eating and living healthy, but also because this lack of a healthy lifestyle so early may trigger the activation of latent genes more quickly. The cause of T2D varies from person to person, and can be due to a combination of genetic factors and lifestyle choices. It can, however, be controlled more easily when the body is kept healthier. Beside altered gene expression, misguided intracellular signaling in various body tissues have also been implicated in T2D. Pancreatic beta cells are especially susceptible to damage by high blood sugar, and since these same beta cells produce the insulin that controls blood glucose levels, additional damage to these cells creates a vicious circle that only exacerbates the situation. The liver also has a large role in T2D development. Normally the liver releases glucose at times of low blood sugar in response to glucagon produced by the pancreas. Once sugar levels are normalized, the liver no longer releases glucose stores until the body needs it again. However, in the diabetic condition, the liver may continue to release glucose even after sufficient levels are present in the blood stream. The net result is excessively high blood sugar levels which can cause more damage to the liver or pancreatic beta cells.

### 3.3 Linkage of Type 2 Diabetes to Alzheimer's Disease

Numerous studies have documented a strong association between diabetes and Alzheimer's Disease (AD) [16], [17], [18], [19], [20], [44]. However, the mechanisms underlying this association have not been clearly established. Studies have shown that insulin resistance or deficiency alters Aβ and tau protein phosphorylation which lead to the onset of AD [45]. Some researchers have proclaimed that AD is really a form of “Type 3 Diabetes” [46]. Insulin resistance in the brain typically precedes and contributes to cognitive decline above and beyond other known causes of AD [47] and abnormalities in the activity of two major signaling pathways for insulin and insulin-like growth factor in non-diabetic people with AD has been identified [48]. Thus, brain insulin resistance appears to be an early and common feature of AD. These insulin signaling pathways could be targeted with new or existing medicines to potentially help brain insulin resistance and possibly reduce or even improve cognitive decline. Butyrylcholinesterase and acetylcholinesterase were commonly found in high levels in both AD and diabetic patients which may play an etiological role influencing insulin resistance and lipid metabolism. Alpha7 nicotinic acetylcholine receptor is an important factor of the cholinergic nerve system in the brain for AD [49]. Antagonists of this receptor, such as the drug memantine, are suitable for the treatment of AD. There may be a potential role of the alpha 7 nicotinic acetylcholine receptor in reducing inflammatory neurotoxicity in AD [50]. Zinc deficiency is commonly noted in both AD and T2D [31], [51]. Moreover, zinc is critical in the enzymatic non-amyloidogenic processing of the amyloid precursor protein (APP) and in the enzymatic degradation of Aβ peptide. On the other hand, zinc binds to Aβ which promotes its aggregation into a neurotoxic species, and as a result, disruption of zinc homeostasis in the brain results in synaptic and memory deficits. Thus, zinc dyshomeostasis may play a critical role in the pathogenesis of AD [52]. Furthermore, these authors proposed that chelating zinc or regulating zinc is a potential AD therapeutic treatment. Zinc and nutritional supplements of zinc showed beneficial effects on diabetes [53]. More importantly, both AD and T2D have insulin degrading enzyme (IDE) deficiency, and increasing IDE synthesis is beneficial for both AD and T2D as discussed in the next chapter.

## 4 Clinical impact of insulin degrading enzyme (IDE) on Alzheimer's Disease and Type 2 Diabetes

Human insulin degrading enzyme (IDE) was first discovered in 1988 [54]. The IDE gene is located on chromosome 10q23–24 in humans, and its defect affect AD pathogenesis [55]. Only recently has it been discovered that IDE cleaves both insulin and amyloid-β and therefore may be linked to both AD and T2D [56], [57]. More recently, a zinc-dependent metalloprotease, neprilysin (NEP) that degrades both Amyloid β peptides 1–40 and 1–42 has been identified [58]. Although it has many other substrates such as enkephalin, atrial natriuretic peptide, endothelin, and substance P, it is not known to degrade insulin. Although there are many publications discussing the importance of IDE activities on Alzheimer's Disease (AD) and Type 2 Diabetes (T2D), interpretation of these data on the treatment of diabetes is controversial or confusing. In fact, stimulation of IDE synthesis in the body is helpful for the prevention and treatment of both T2D and AD. Cognitive impairment and T2D are common disorders in the elderly. However this co-existence could be a scenario in which T2D and cognitive impairment share causal pathways, or cognitive impairment could be implicated in T2D, or vice versa [59]. The exact mechanism that leads T2D to cause cognitive dysfunction and dementia, or conversely, the role of AD on the induction of diabetes has not been clearly established. It is known that hyperglycemia and insulin resistance can cause structural changes in the hippocampus and hypothalamus, which are involved in regulating carbohydrate metabolism and branched chain amino acid homeostasis, which are impaired in patients with AD [60]. A clear relationship between AD and T2D has been established by the fact that mice with induced IDE deficiency exhibited both AD and T2D symptoms [34], [35] and similarly findings were reported in humans [30], [61], [63].

Many of the diabetes studies indicate that inhibition of extracellular IDE activities may be a useful method for treating diabetes [64], [65], since extracellular IDE can interfere with normal blood glucose transport by decreasing plasma insulin levels within diabetic subjects. Although, plasma insulin levels decrease to a certain amount by increasing extracellular IDE, it is the level of cytosolic IDE synthesis, not extracellular levels, that is important for the treatment of insulin resistance in diabetic subjects. Intracellular IDE degrades inactive internalized insulin preventing the accumulation of incompletely degraded insulin fragments in the cytosol which can interfere with the insulin signal transduction mechanism for glucose uptake. IDE therefore improves insulin sensitivity by removing internalized insulin and insulin-related degradation peptides. Thus, IDE synthesis stimulation is beneficial for the prevention and treatment of both AD and T2D.

### 4.1 Cyclo-Z treatment for IDE synthesis

Many publications indicated that plasma zinc levels in patients with Alzheimer's Disease (AD) significantly decreased compared to controls [66], [67], [68] and in patients with Type 2 Diabetes (T2D) [33]. Since IDE is a zinc requiring enzyme, treatment with zinc supplementation would be warranted. In fact, many studies indicated that zinc homeostasis is an important factor for the prevention and treatment of AD. However, others indicated that zinc may stimulate Aβ aggregation causing more harmful in AD [70], [71]. These conflicting reports were explained by the fact that 1 mM dose of zinc may be harmful for AD patients as zinc may reinforce Aβ aggregation and result in an increase in the number or size of neuronal Aβ plaques while low zinc (50 μM) exerts protective effects against Aβ-40 induced toxicity [72]. Zinc deficiency is mostly caused by impaired intestinal zinc absorption and cellular zinc uptake. Cyclo(His-Pro) has a chelating effect on zinc and assists in zinc transport through an intestinal Cyclo(His-Pro) transporting mechanism independent of the normal elemental zinc transport system [73]. The normal elemental zinc absorption mechanism is a facilitated diffusion transport process, via the zinc transporter (ZnT) proteins mediating zinc efflux and influx into intracellular vesicles [74]. These proteins contain a variable number of histidine, similar to Cyclo(His-Pro). Human serum albumin has a stronger inhibitory effect on zinc medicated Aβ-42 fibrillogenesis and cytotoxicity [75]. Evidence from these studies for inhibiting zinc mediated amyloid β-protein fibrillogenesis and cytotoxicity by serum albumin support the hypothesis that Cyclo-Z, which contains dipeptides similar to serum albumin may be helpful in preventing and treating AD, based on the fact that Cyclo-Z contains high amounts of histidine to chelate zinc and thus stimulate intestinal zinc absorption.

**Fig. 1:**
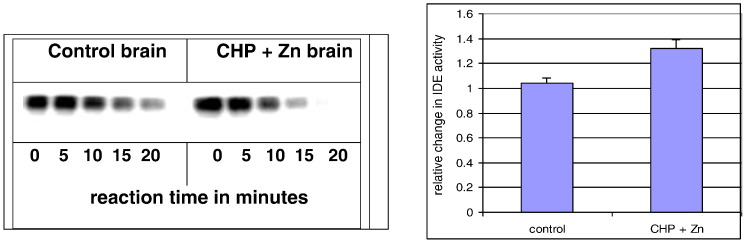
Enzymatic activity on insulin degradation in Cyclo-Z treated mouse brain. To understand the mechanisms by which Cyclo-Z affects insulin sensitivity, we tested the effects of adding 10 mg zinc plus 1.0 mg CHP/L to the drinking water on IDE activity in the brain tissues of APP transgenic mice. IDE in the cytosol of brain samples from control animals requires more than 20 min to degrade radiolabeled insulin whereas the cytosol from Cyclo-Z treated animals degraded 100% of the insulin within 15 min (left). This is approximately a 30% enhancement of IDE activity by Cyclo-Z therapy (right) (n = 6). IDE is the only known enzyme that can digest both insulin and amyloid protein; and since zinc is an integral part of IDE, it is absolutely required for IDE enzyme activity. Hence, Fig. 1 supports the hypothesis that Cyclo-Z treatment may ameliorate diabetes by increasing IDE synthesis in muscle or brain cells.

**Fig. 2:**
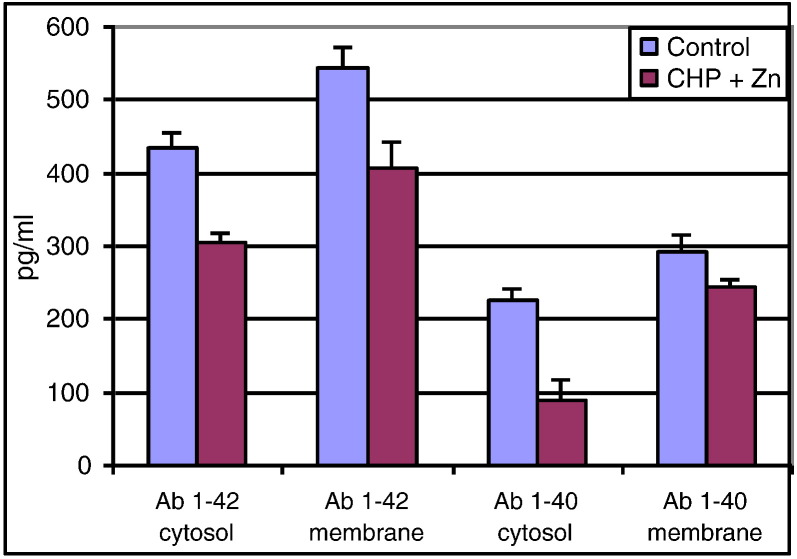
Levels of Aβ 1–40 and 1–42 in brain cytosol and membrane fractions in Cyclo(His-Pro) (CHP) plus zinc treated mouse brain. Brain fractions were analyzed for levels of Aβ 1–40 and 1–42 by ELISA after treatment with CHP + zinc or control (n = 6 mice per group). These studies demonstrate that CHP plus zinc treatment with 1.0 mg/ml CHP and 10 mg/L Zn in their drinking water for 5 weeks decreases Aβ 1–40 and 1–42 levels in transgenic mice expressing human APP. CHP plus zinc treatment caused a 60% reduction in cytosolic Aβ 1–40 and a 25% reduction in Aβ 1–42 over this time period.

### 4.2 IDE action on the prevention and treatment of AD

The etiology of AD is still not clearly understood, and no safe and effective anti-AD drug to prevent, halt, or reverse the progression of AD is currently available. Insulin degrading enzyme (IDE) and Neprilysin (NEP) has been known to be the only known enzymes that degrades Aβ40, Aβ42 [58]. The IDE gene is located on chromosome 10q23.3 close to a region of linkage for the late-onset Alzheimer's Disease susceptibility genome [79]. A mouse model of AD shows decreased IDE levels in the cerebrum and accelerated phenotypic features of AD [80]. Through immunohistochemical studies, this group also demonstrated that human astrocytic phospholipase A2 group 3 (Pla2g3) expression was significantly increased in human AD brains compared to controls. One function of Plag2g3 is to induce chronic oxidative stress, which is highly relevant to the slow progression of AD and the reduction of IDE The authors concluded that the increase of Pla2g3 expression contributes to decreased levels of IDE and suggested that Plag2g3 is involved in the initiation and/or progression of AD. Others have shown that upregulation of Neprilysin (NEP) also reduces Aβ accumulation in the brain [81]. NEP, a zinc-dependent metalloprotease similar to IDE, cleaves and inactivates several peptide hormones including neurotensin, bradykinin, and neuropeptide FF [82]. IDE and NEP, both zinc enzymes, have comparable effects for clearing Aβ with similar catalytic action [83]. However, somatostatin treatment preferentially upregulates IDE, compared to NEP [81]. Somatostatin is a peptide hormone that regulates the endocrine system and affects neurotransmission and cell proliferation via interaction with G protein-coupled somatostatin receptors and inhibition of the release of numerous secondary hormones. It is possible that zinc supplementation may stimulate synthesis of both IDE and NEP.

### 4.3 IDE action on the prevention and treatment of Type 2 Diabetes

**Fig. 3:**
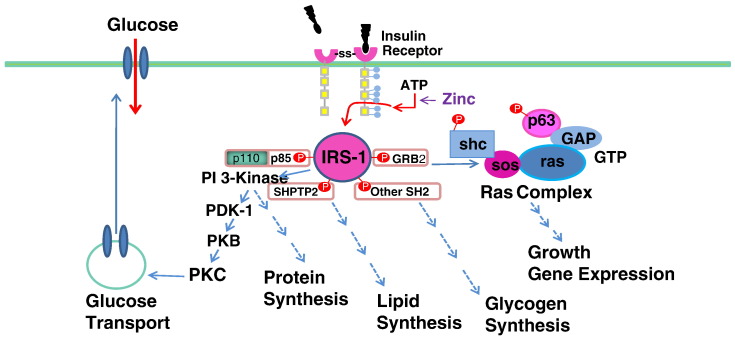
Signaling pathways involved in insulin-dependent glucose uptake, gene expression, or enzyme synthesis. Circulating insulin secreted by pancreatic B-cells first binds to the insulin receptor α-subunit at the cell membrane surface followed by signaling to the β-subunit which is auto-phosphorylated. In the absence of insulin treatment, zinc will activate ATP to phosphorylate the β-subunit of the receptor. Then, the β-subunit of the receptor will also be auto-phosphorylated. The phosphorylated insulin receptor initiates a cascade of phosphorylation events: Receptor Subunit-1 (IRS-1); phosphoinositol 3′-kinase (PI 3′-kinase); 3-phophoinositide-dependent protein kinase-1 (PDK-1); protein kinase B (Akt/PKB); atypical Protein kinase C (PKC). This cascade activates glucose transporter synthesis and/or translocation.

**Fig. 4:**
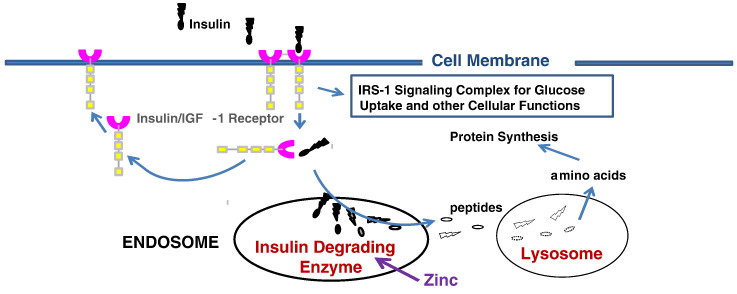
Cytosolic degradation of insulin. After the insulin-receptor mediated signal transduction process is initiated, the inactive cellular insulin must be degraded in the endosome by insulin degrading enzyme (IDE). The degraded insulin fragments will be completely digested into amino acids through lysosomal proteases like Cathepsin D. IDE levels in the cytosol are reduced in diabetic subjects probably due to zinc deficiency.

### 4.4 Metabolic association of IDE between Alzheimer's Disease and Type 2 Diabetes

Alzheimer's Disease (AD) and Type 2 Diabetes (T2D) are closely related to each other as both exhibit common metabolic disorders including zinc [90], [91] and insulin degrading enzyme (IDE) deficiencies [30], [35]. Mittal et al. [56] has also suggested that AD is a neuroendocrine disorder and that IDE could be the major player which possesses the ability to shift T2D to other metabolic pathways such as regulation of amyloid beta degradation by IDE. T2D and AD share the pathological characteristics of amyloid deposits derived primarily from islet amyloid polypeptide (APP) in T2D β-cells and amyloid β (Aβ) in AD neurons [57], [92]. There has been disagreement in the research community regarding the interpretation of the role of IDE in the treatment of diabetes. Based on the ability to lower plasma insulin levels, many researchers concluded that IDE inactivation or inhibition of IDE synthesis may help in the control of blood glucose metabolism [64], [65]. However, more recently, researchers have reported that IDE activity is essential to maintain normal insulin sensitivity in humans [37], [94]. We also agree with the latter group that IDE stimulation is beneficial for the treatment of both AD and T2D.

## 5 Methods of treating Alzheimer's Disease and Type 2 Diabetes

### 5.1 Treatment of Alzheimer's Disease

**Fig. 5:**
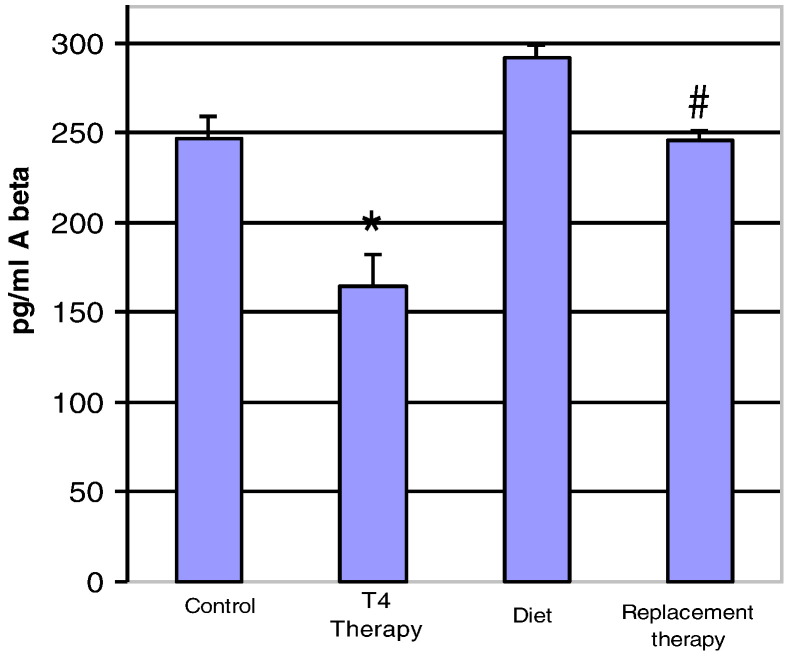
Aβ 1–42 levels in hyperthyroid and hypothyroid mouse brain. Mice were first made hypothyroid for three weeks with an iodine-deficient PTU diet, and then replaced with T_4_ to reverse hypothyroidism. *P < 0.05: Aβ levels in T4 treated mice were compared to controls. ^#^P < 0.05: T4 replaced mice in hypothyroid animals were compared to non-treated hypothyroid animals.

**Fig. 6:**
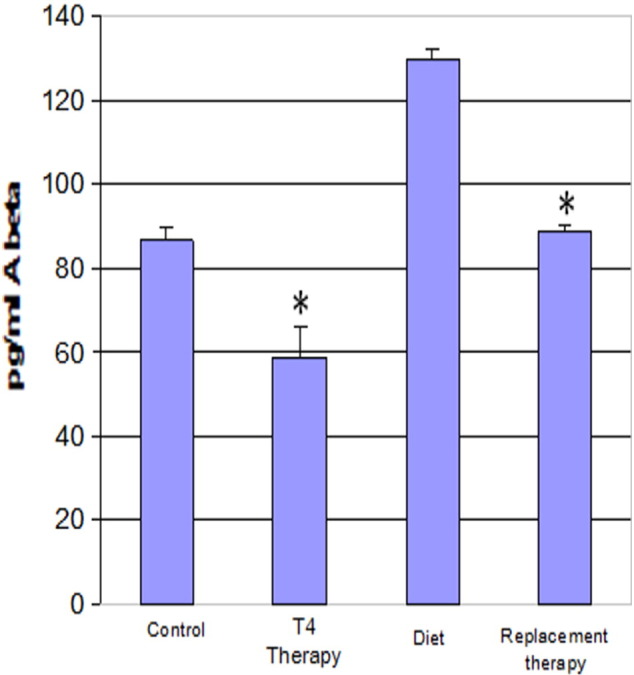
Aβ 1–40 levels in Hyperthyroid and Hypothyroid Mouse Brain. *P < 0.05: Aβ levels in T4 treated mice were compared to controls. ^#^P < 0.05: T4 replaced mice in hypothyroid animals were compared to non-treated hypothyroid animals.

#### 5.1.1 Treatment of Alzheimer's Disease with zinc plus Cyclo(His-Pro) plus Zinc

In contrast to IDE effects in the treatment of diabetes, IDE effects on Alzheimer's Disease (AD) are well established. IDE has been shown to degrade circulating Aβ and decrease plaque formed Aβ deposits in brain cells [30], [63] and minimizing plasma Aβ protein levels, either before or after Aβ plaque formation by increasing IDE activity in the blood, would be highly desirable in AD treatment. This could be done by increased IDE synthesis; however, no drugs are currently available that augment IDE levels. It is known that AD [31], [99] and cognitive impairment [31], [100] exhibit a positive correlation with zinc deficiency, and zinc supplementation may help in reducing cognitive impairment [100], [101] and has a role in the control of the inflammatory response [102]. However, some have reported that large doses of zinc can worsen AD symptoms by binding to and inducing aggregation of Aβ [72]. Thus, treatment of AD patients with zinc has been avoided. However, a recent report disputed this conclusion by demonstrating that: 1) copper is frequently a toxic trace element in zinc preparations that exacerbated AD, 2) zinc is a neuronal protective factor for AD, and that 3) zinc deficiency is the actual cause of AD progression [31]. Since AD plaque burden starts accumulating 20 years before the onset of the disease, adequate early zinc intake may be a protective factor to slow or prevent the development of AD [101]. It has been proven that zinc therapy significantly improved patients' cognitive abilities [99], [100], [101], [102], [103], and therefore it is recommended that patients consume the Recommended Daily Allowance (RDA) of zinc without overdosing. Thus, maintaining zinc homeostasis is highly desirable for the prevention and possible treatment of AD.

**Fig. 7:**
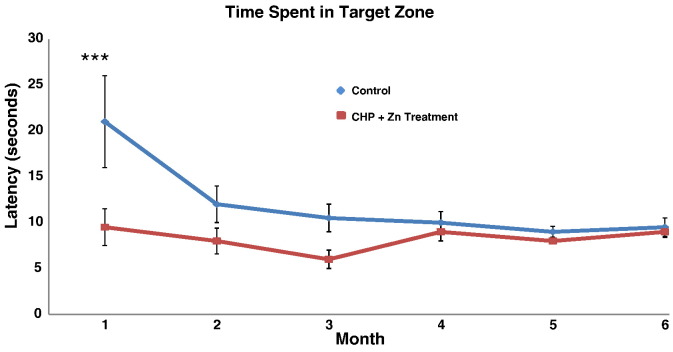
CHP plus zinc treatment enhances spatial memory in huAPP transgenic mice. HuAPP transgenic mice (9 months of age) were divided into 2 groups of 3 animals each and treated as follows: control group, animals were given access to H_2_O alone ad libitum; CHP plus zinc group, animals were given access to 1.0 mg/L CHP + 10 mg/L Zn in H_2_O ad libitum. Once a month, the animals were subjected to the Morris Water Maze to measure the time it took for the animal to find the platform. Very significant improvement in spatial memory is seen in one month in the CHP plus zinc treated mice. ***P < 0.001 for average ± SEM between CHP plus zinc treatment group and control group by Tukey multiple comparison statistical tests.

### 5.2 Treatment of diabetes

#### 5.2.1 Insulin action on glucose metabolism

Circulating insulin secreted by pancreatic β-cells binds to the insulin receptor α-subunit, followed by signaling of the β-subunit to be auto-phosphorylated [Fig. 3]. In the absence of insulin signaling, zinc will activate ATP to phosphorylate the β-subunit of the receptor [104]. The phosphorylated insulin receptor then initiates a cascade of phosphorylation events: first, Insulin Receptor Subunit-1 (IRS-1), followed in succession by Phosphoinositol 3′-kinase (PI 3′-kinase) 3-Phophoinositide-Dependent Protein Kinase-1 (PDK-1), Protein Kinase B (Akt/PKB), and then atypical Protein Kinase C (PKC). The result of this insulin-dependent phosphorylation cascade is glucose translocation and/or glucose transporter synthesis [105]. In addition to its role in glucose metabolism, insulin also affects cell growth, gene expression, and the synthesis of proteins, lipids, and glycogen. After the induction of this signal transduction mechanism for glucose uptake, the insulin-receptor complex is internalized. To reset the system, the inactive insulin is separated from the receptor, and the receptor is either recycled to the cell surface or degraded in the cytosol. In the meantime, insulin is degraded by IDE in the endosome [106], [107] and then completely digested into amino acids by lysosomal peptidases. If insulin is incompletely degraded in the cytosol, insulin peptide fragments in the cytosol can interfere with insulin-mediated signal transduction mechanisms resulting in insulin resistance and diabetes. In diabetic subjects, IDE levels in the cytosol are reduced and consequently more and more IDE synthesis is needed for complete insulin degradation. Support for this mechanism of insulin resistance is indicated in the fact that inhibition of IDE with BDM44768 resulted in impaired glucose tolerance [85]. However, others have reported that IDE deficiency actually improved glucose tolerance [108] and based on this, some researchers are now proposing the use of IDE inhibitors as an anti-diabetes agent [86]. Deprez-Poulain et al. [85] reported that IDE is involved in pathways that modulate short-term glucose homeostasis, but casts doubt on the general usefulness in the inhibition of IDE to treat diabetes. The effect of IDE levels on blood glucose control is therefore controversial. The controversy stems from a fundamental misunderstanding of the differing roles of extracellular plasma vs. intracellular IDE activity. Extracellular IDE degrades active insulin molecules in the plasma. Increased IDE activity at the extracellular level may be detrimental for blood glucose control since it reduces plasma insulin levels and promotes insulin resistance. IDE inhibition (extracellular) prevents this decrease in plasma insulin levels, and therefore more plasma insulin results in increased glucose transport in diabetics. On the other hand, intracellular IDE is involved in the degradation of internalized inactive insulin after the initiation of the glucose transporter translocation pathway for glucose uptake (Fig. 4). Internalized insulin degradation by IDE is useful for the prevention and treatment of diabetes as we have observed since it degrades the insulin-insulin receptor complex and resets the insulin signaling mechanism.

#### 5.2.2 Treatment of Type 2 Diabetes with Cyclo-Z

**Fig. 8:**
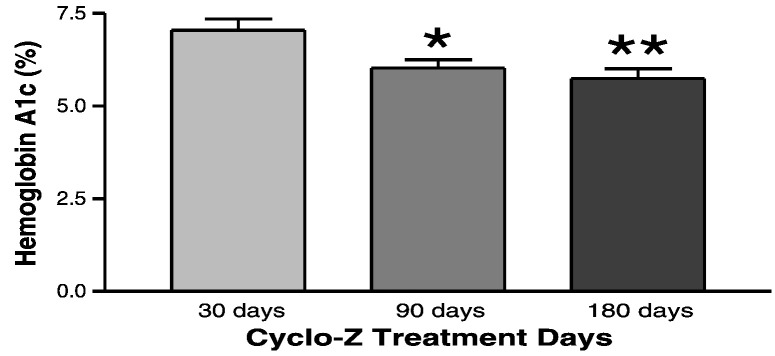
CHP plus zinc treatment decreases Hemoglobin A1 levels in diabetic subjects. In a small scale clinical trial, diabetic patients (n = 18) were treated with CHP plus zinc and Hemoglobin A1 (HbA_1c_) levels were determined at 30, 90, and 180 days. Significant reduction in HbA_1c_ levels are seen at 90 and 180 days in patients treated with CHP plus zinc. *P < 0.05; **P < 0.01 vs. 30 days.

**Fig. 9:**
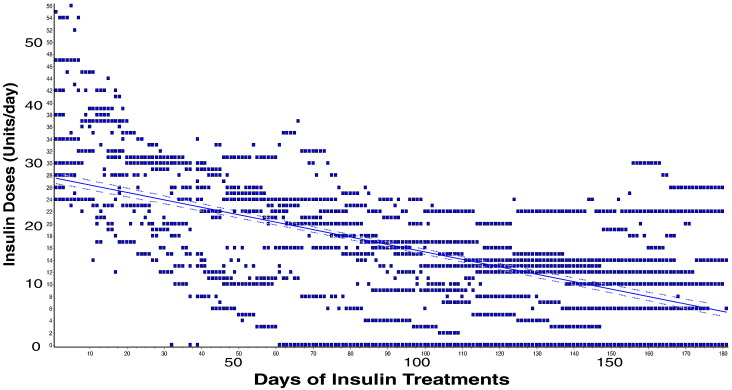
CHP plus zinc treatment decreases required insulin doses needed to stabilize glucose levels in diabetic subjects. Diabetic patients (n = 18) treated with CHP plus zinc for 180 days required significantly less insulin daily to maintain healthy levels of glucose. The decreasing rate of total insulin injection per day was − 0.1126 ± 0.0042 units/day. P < 0.0001.

**Table 1:** Mean monthly average blood glucose level changes during six-months Cyclo-Z treatment period.

mmol/L blood glucose				
Treatment days	Fasting mean ± SEM	After breakfast mean ± SEM	After lunch mean ± SEM	After dinner mean ± SEM
− 15–0	7.23 ± 0.31	10.59 ± 0.64	10.63 ± 0.48	10.29 ± 0.72
0–30	7.27 ± 0.33	9.53 ± 0.34	9.57 ± 0.30⁎⁎⁎	9.07 ± 0.34
30–60	8.12 ± 0.47	8.80 ± 0.34⁎	8.53 ± 0.23⁎⁎⁎	8.55 ± 0.30⁎
60–90	7.52 ± 0.32	8.21 ± 0.27⁎⁎⁎	8.05 ± 0.30⁎⁎⁎	7.85 ± 0.25⁎⁎⁎
90–120	7.11 ± 0.29	7.84 ± 0.30⁎⁎⁎	7.86 ± 0.27⁎⁎⁎	8.01 ± 0.28⁎⁎
120–150	7.08 ± 0.32	8.28 ± 0.29⁎⁎⁎	8.19 ± 0.33⁎⁎⁎	8.16 ± 0.31⁎⁎
150–180	7.46 ± 0.42	8.79 ± 0.37⁎	8.31 ± 0.34⁎⁎⁎	8.39 ± 0.32⁎

⁎ P < 0.05.

⁎⁎ P < 0.01.

⁎⁎⁎ P < 0.001.

### 5.3 Treatment relationship between Alzheimer's Disease and Type 2 Diabetes with Cyclo-Z

The etiology of Type 2 Diabetes (T2D) and Alzheimer's Disease (AD) are similar to each other [46]. In fact, brain insulin resistance develops before the onset of AD symptoms [47]. Brain tissues require a high level of glucose energy intake (about 20%) in humans and mammals and brain energy deficiency might be a contributing factor of abnormal brain function in AD [112]. However, the most plausible common factor between AD and T2D is deficiency of IDE activity due to zinc deficiency [31], [33]. Zinc is an integral part of IDE, which is a zinc protease, involved in both IDE gene expression and required for IDE activity. Thus, Cyclo-Z is expected to be a successful treatment for both T2D and AD as it functions to transport zinc into the cells. Both zinc and CHP increased insulin secretion (secretagogues) and plasma insulin levels without improving insulin sensitivity in muscle and fat cells. In addition, Cyclo-Z increased IDE synthesis [Fig. 1] and reduced both harmful amyloid-β proteins in the brains of AD-induced mice [Fig. 2]. These facts suggest that Cyclo-Z treatment is an excellent potential candidate in the treatment of both AD and T2D patients.

## 6 Treatment strategy

### 6.1 Alzheimer's Disease diagnosis

Prior to the treatment of disease, it is essential to have a correct and proper diagnosis differentiating Alzheimer's Disease (AD) from other memory and behavioral disorders. Unfortunately, using even the most recent guidelines for diagnostic criteria, early stages of AD are not easily identified. Core clinical criteria for later stage AD dementia include insidious onset and worsening cognition, amnesic presentation, worsening language presentation, difficulty of visual-spatial presentation, and executive dysfunction [113]. Pathological evidence is seen in lower levels of Aβ42 protein in the cerebrospinal fluid and increased deposition of Aβ in brain plaques and tau in neurofibrillary tangles. However, Aβ deposition and elevated levels of tau proteins are also shown in other neurodegenerative disorders such as amyloid angiopathy and prion diseases [114] and are frequently only identified at the time of death. Researchers are trying to identify early biomarkers of AD that may signal when pre-symptomatic brain change occurs using non-invasive techniques such as magnetic resonance imaging (MRI) and position emission tomography (PET) or through analysis of cerebrospinal fluid (CSF) proteins such as amyloid beta and tau. Plasma IDE levels might be an effective and new plasma biomarker for AD pathological progression for which further research is warranted.

### 6.2 Alzheimer's Disease treatment

**Table 2:** Alzheimer's Disease treatment medications approved by US FDA.

Drug description	Trade name	Approved for	Biochemical actions	Side effects
Donepezil	Aricept	All stages	Inhibiting hydrolysis of acetylcholine	Nausea, vomiting, loss of appetite and increased frequency of bowel movement
Galantamine	Razadyne	Mild to Moderate	Inhibiting hydrolysis of acetylcholine	Nausea, vomiting, loss of appetite and increased frequency of bowel movement
Memantine	Namenda	Moderate to severe	Blocking the activity of the neurotransmitter glutamate	.Headache, constipation, confusion and dizziness
Rivastigmine	Exelon	All stages	Inhibiting hydrolysis of acetylcholine and butyrylcholine	Nausea, vomiting, loss of appetite and increased frequency of bowel movement
Donepezil and memantine	Namzaric	Moderate	Dual activities: inhibiting hydrolysis of acetylcholine and blocking the neurotransmitter glutamate	Nausea, vomiting, loss of appetite and increased frequency of bowel movement, headache, constipation, confusion, and dizziness

The second mechanism targeted for AD treatment is the inhibition of *N*-methyl-d-aspartate receptors (NMDD) which regulates the activity of glutamate, a neurotransmitter in the brain involved in learning and memory. Drugs such as memantine and galantamine are NMDD antagonists that block the activity of the *N*-methyl-d-aspartate (NMDA) receptors. The general mechanisms of acetylcholinesterase inhibitor (AChEI) is to increase the brain availability of acetylcholine by inhibiting acetylcholinesterase activity [118]. Another strategy for the treatment of AD is blocking abnormal glutamatergic neurotransmission. Memantine has been characterized as an uncompetitive voltage-dependent NMDA receptor antagonist, with moderate binding affinity, and rapid blocking-unblocking receptor kinetics. For this reason, memantine has been indicated for the treatment of patients with moderate to severe AD [119]. It has shown a significant efficacy in the treatment of AD in several large-scale, placebo controlled studies.

### 6.3 Type 2 Diabetes diagnoses

Early Type 2 Diabetes (T2D) symptoms and signs include fatigue, increased thirst and urination, blurry vision, and slow wound healing. Since these symptoms gradually develop, the patient may not be aware of them until they become quite severe. A T2D screening test is recommended for those who are overweight, have a sedentary lifestyle, have a family history of T2D, or history of gestational diabetes. Clinical tests measure Hemoglobin A1c (HbA1c), fasting plasma glucose, random plasma glucose, and oral glucose tolerance. HbA1c levels above 6.5% are considered diabetic, between 5.7 and 6.4 are considered to be pre-diabetic, and below 5.7 are normal. Clinical levels for fasting blood glucose levels above 126 mg/dL indicate diabetes, between 100 and 125 mg/dL indicate pre-diabetes, and below 100 mg/dL are normal. Values above 200 mg/dL indicate diabetes, between 140 and 199 mg/dL indicate pre-diabetes, and less than 140 mg/dL are normal for random blood glucose testing. In the oral glucose tolerance test (OGGT) analysis, the patient fasts overnight and then consumes a 75 gram glucose solution in the morning. Blood glucose levels are measured 2 h after consumption of the glucose solution. Patients with blood glucose levels above 200 mg/dL are considered diabetics, between 140 and 199 mg/dL are considered pre-diabetics, and below 140 mg/dL are normal. A modification of the OGGT procedure known as the Three hour Average area-above Fasting Glucose Concentration (TAFGC) gives the most accurate OGGT measurement. In order to measure TAFGC, the patient drinks a 75 g glucose solution and then blood glucose levels are measured every 30 min for 3 h. The data is graphed and the area under the curve is integrated. These values are an indication of the pathophysiology of the disease and the efficacy of treatment since low values correlate with an improvement of diabetes pathology.

### 6.4 Type 2 Diabetes treatment

The most ideal treatment for Type 2 Diabetes (T2D) diabetes is lifestyle modification that would improve insulin sensitivity. Diabetes and obesity are adult onset metabolic diseases which are potentially curable diseases if one is highly motivated. It has been reported that lifestyle modification based on physical and/or dietary intervention improved 2 hour plasma glucose measurement (OGTT) and fasting blood glucose levels in patients with impaired glucose tolerance [120]. Lifestyle modification plans are as below:

#### 6.4.1 Diet plan to control

##### 6.4.1.1 Diabetes and obesity

To maintain blood glucose and body weight control for every participant, patients should follow diet instructions to include the following themes: 1) nutrition, 2) menu planning, 3) use of an exchange list, and 4) methods of carbohydrate counting. General dietary instructions will include: (i) eating meals and snacks at about the same time each day; ii) eating a variety of foods to get the vitamins and minerals needed; (iii) intake of 2000–2400 cal daily for a large sized man; (iii) intake of 1200–1600 cal daily for a medium sized woman. Detailed information for the control of body weight should be determined with the help of a clinical dietitian or by the patient's primary physician.

#### 6.4.2 Activity plan to maintain glucose and weight control

Exercise for diabetes and obesity is very critical, since exercise is the single best predictor of long-term blood glucose and weight control. In accordance with national guidelines, moderately intense physical activity is recommended for at least 30 min on 5 or more days per week. These activities can include any of the following: 1) walking briskly, 2) mowing the lawn, 3) dancing, 4) swimming, 5) bicycling, 6) and other activities with similar energy expenditure. Physical activity is started slowly, and subjects are encouraged to increase their activity until they have reached a goal of at least 30 min of activity 5 or more times per week.

#### 6.4.3 Record keeping methods

Based on the patients' daily records, dietary intake, type of food, and calorie intake can be estimated. Implementation of recording keeping is very important to keep the subjects compliant with the planned program: 1) by examining their accomplishments, 2) by documentation of the progress of reduced body weight, and 3) by developing a habit of a healthy modified life style.

#### 6.4.4 Record keeping of drug intake

**Table 3:** Classification of anti-diabetes agents for Type 2 Diabetes.

Drug class	Drug trade name	Drug description	Biochemical actions
Improve insulin-resistance	Avandia, Actos	Rosiglitazone, pioglitazone	PPARγ activators
	Duvie	Lobeglitazone	
	Cyclo-Z	Cyclo(His-Pro) plus zinc	IDE synthesis stimulator
Increase pancreatic insulin secretion	Sulfonylurea derivatives (SD) 1st Generation	Carbutamide, Acetohexamide Chlorpropamide, Tolubutamide	Increase insulin release from the pancreatic β-cells by cell depolarization to enhance influx of Ca^+ 2^ and efflux of K^+ 1^ ions
	SD 2nd Generation	Glyburide, Glipizide, Gliclazide Glibenclamide, Glibornuride, Gliquidone, Glisoxepide, and Glyclopyramide	
	SD 3rd Generation	Glimepiride (Amaryl)	
	Phenylalanine derivatives	Meglitinides, Nateglinide, Repaglinide (Prandin)	Similar to sulfonylurea derivatives by closing ATP-dependent K^+ 1^ and opening Ca^+ 2^ channels of β-cells to increase insulin secretion
	Glucagon like Peptide-1 (GLP-1)	Tanzeum, Victoza	GLP-1 signals pancreatic β-cells to secrete insulin
	Dipeptidyl peptidase-4 (DPP-4)	Sitagliptin, Januvia	DPP-4 inhibits degradation of GLP-1 which increases insulin secretion of β-cells
Inhibition of hepatic glucose production	Biguanides	Metformin, Glucophage, Fortamet	Inhibition of glycogen to glucose conversion (glucogenesis) in the liver
Inhibition of intestinal glucose absorption	Acarbose	Precose, Glucobay, Prandase	Inhibition of alpha-glucosidase reducing intestinal glucose absorption
	Miglitol	Migliol	
Increase plasma insulin levels	Rapid-acting insulin	Rapid-acting insulin	Supplemental plasma insulin
	Novolog (70% insulin aspart protamine suspension and 30% insulin aspart injection mixture)	Moderate acting insulin	
	Levemir (insulin detemir) human insulin analogue (rDNA orgin)	Long acting insulin	
Inhibit renal glucose reabsorption	Gliflozins Dapagliflozin (Farxiga), Canagliflozin (Invokana)	Inhibitor of sodium-glucose co-transporter 2 (SGLT2)	Increase urinary glucose secretion

## 7 Conclusions

Alzheimer's Disease (AD) is sometimes referred to as “Type 3 diabetes” since many of the clinical abnormalities of Alzheimer's Disease are similar to diabetes. Diabetics have impaired cognitive performance compared to age-matched control subjects, but the pathological basis for this impairment is largely unknown [121]. The prevalence rates for AD with T2D were 4.51% and 3.65% for vascular dementia [122], while it was 3.12% of the general population of the US in 2012 [123]. On the other hand, incidence of diabetes in AD patients was about 20% in the US [112]. It is reported that T2D may be a major risk factor for AD in the absence of other known major AD risk factors [124], [125]. Although the exact physiochemical factors linking AD to T2D remains uncertain, there yet appears to be a strong physiochemical relationship between AD and T2D [56]. Based on the current literature, IDE activity is the most plausible relationship between AD and T2D [25], [44], [56], [126], [127]. The majority of IDE literature indicated that homeostasis of zinc and IDE metabolism is the common metabolic abnormality between AD and T2D, and that IDE regulation may be essential for treatment of AD and T2D. However, current drug treatment of these two conditions are vastly different from each other. Most drug treatments for AD subjects are cholinesterase inhibitors while diabetes treatments mostly involve the use of insulin secretagogues, such as sulfonylurea derivatives, or injectable insulin for blood glucose control. Only thiazolidinedione derivatives improve insulin resistance and have the best potential for curing diabetes. However, thiazolidinedione derivatives as well as the other drug treatments for AD and T2D have severe side effects. The only common link between AD and T2D is zinc and IDE deficiency. Thus, a preferred treatment method for both AD subjects and T2D subjects would be uptake of zinc for stimulation and activation of IDE synthesis. The importance of IDE in AD is well established as IDE is the only known enzyme that degrades β-amyloid proteins, which are a major cause of AD pathology, and that degrades used insulin which is the cause of insulin resistance. Both extracellular and intracellular IDEs are beneficial for the treatment of AD subjects. However, the role of IDE for T2D is controversial. Most researchers believe that IDE is harmful for diabetes and that an IDE inhibitor is an ideal candidate for the treatment of T2D. These scientists are only thinking about extracellular IDE, which degrades insulin circulating in the blood. Extracellular IDE may indeed be detrimental for blood glucose control since extracellular insulin is needed for blood glucose transport and IDE degrades insulin. This appears to be a credible theory, as many researchers have suggested. However, in contrast to extracellular IDE, intracellular IDE is a vital factor for the improvement of insulin sensitivity.

When insulin is bound to the insulin-receptor, it signals the glucose transporter to translocate to the cell membrane and open the glucose entry gate for glucose uptake by the cells. After its role in signaling is complete, the insulin-receptor complex becomes internalized, and insulin and its receptor are separated. The receptor is recycled or destroyed by lysosomal enzymes. However, the used insulin must be degraded by IDE via the endosome before lysosomal proteases can digest the insulin fragments completely into amino acids. When IDE levels are reduced in the cytosol, incompletely degraded or inactive insulin remains in the cytosol and interferes with insulin signal transduction mechanisms resulting in insulin resistance and diabetes. T2D patients are afflicted with deficiencies of both zinc and IDE. Since reduced IDE in the cytosol is not able to degrade all the internalized insulin, increased intracellular IDE would be beneficial for the improvement of diabetes. Cyclo-Z is the only agent able to increase intracellular IDE. In our studies [77], [78] of Cyclo-Z treatment of genetically T2D G-K rats and obese ob/ob mice, plasma insulin levels decreased, while blood glucose decreased and insulin sensitivity increased. Therefore, we conclude that Cyclo-Z treatment increases cellular IDE and improves insulin sensitivity of diabetic rodents. Even though plasma insulin levels are slightly decreased, blood glucose levels in the blood also significantly decreased due to increased insulin sensitivity and transport and metabolism of the glucose into the cells. Thus, Cyclo-Z treatment is expected to be very beneficial for both AD and T2D patients and, as a proof of concept, we have initiated a FDA-approved phase 2 clinical trial for obese diabetic subjects and are in the process of initiating a Phase 2 clinical trial for Alzheimer's Diseases. We expect excellent study results for AD and T2D treatments using Cyclo-Z, which is the only chemical known to stimulate IDE synthesis and cause both the increased degradation of amyloid beta proteins and reduction of inactive cellular insulin fragments.

## Transparency document

Transparency document. Image 1
